# Disaster preparedness and response capacity of regional hospitals in Tanzania: a descriptive cross-sectional study

**DOI:** 10.1186/s12913-018-3609-5

**Published:** 2018-11-06

**Authors:** Philip M. Koka, Hendry R. Sawe, Khalid R. Mbaya, Said S. Kilindimo, Juma A. Mfinanga, Victor G. Mwafongo, Lee A. Wallis, Teri A. Reynolds

**Affiliations:** 1 Emergency Medicine Department, Muhimbili University of Health and Allied Sciences, P.O. Box 65001, Dar es Salaam, Tanzania; 2 Emergency Medicine Department, Muhimbili National Hospital, Dar es Salaam, Tanzania; 3 Emergency Department, Al-Zahra Hospital Sharjah, Sharjah, United Arab Emirates; 4 Division of Emergency Medicine, University of Cape Town, Cape Town, South Africa; 5 Department for the Management of NCDs, Disability, Violence and Injury Prevention, World Health Organization (WHO), Geneva, Switzerland

**Keywords:** Disaster preparedness, Africa, Emergency response, Emergency care, Tanzania

## Abstract

**Background:**

Tanzania has witnessed several disasters in the past decade, which resulted in substantial mortality, long-term morbidity, and significant socio-economic losses. Health care facilities and personnel are critical to disaster response. We assessed the current state of disaster preparedness and response capacity among Tanzanian regional hospitals.

**Methods:**

This descriptive cross-sectional survey was conducted in all Tanzanian regional hospitals between May 2012 and December 2012. Data were prospectively collected using a structured questionnaire based on the World Health Organization National Health Sector Emergency Preparedness and Response Tool. Trained medical doctors conducted structured interviews and direct observations in each hospital.

**Results:**

We surveyed 25 regional hospitals (100% capture) in mainland Tanzania, in which interviews were conducted with 13-hospital doctors incharge, 9 matrons and 4 heads of casualty. All the hospitals were found to have inadequate numbers of all cadres of health care providers to support effective disaster response. 92% of hospitals reported experiencing a disaster in the past 5 years; with the top three being large motor vehicle accidents 22 (87%), floods 7 (26%) and infectious disease outbreaks 6 (22%). Fifteen hospitals (60%) had a disaster committee, but only five (20%) had a disaster plan. No hospital had all components of surge capacity. Although all had electricity and back-up generators, only 3 (12%) had a back-up communication system.

**Conclusion:**

This nationwide survey found that hospital disaster preparedness is at an early stage of development in Tanzania, and important opportunities exist to better prepare regional hospitals to respond to disasters.

## Background

Disasters are serious disruptions of the functioning of a community or society, causing widespread human, material, economic and environmental losses that exceed the ability of the affected community or society to cope using its own resources [1, 2]. Disaster preparedness and response include a range of activities to protect communities, property and the environment. Health care facilities are critical to disaster response; they should have a dedicated hospital disaster plan and surge capacity to allow them to quickly expand to accommodate the additional patients affected by a given emergency [3]. Surge capacity is regarded as a marker of the ability to deliver effective emergency care in a disaster situation [4, 5]. Poor disaster preparedness at the hospital level is known to result in poor patient outcomes, provider frustration and fatigue, and overall system disruption [6].

In most high-income countries, disaster preparedness and response are well developed pre-disaster, with clear plans of action established by a team representing multiple sectors [7]. Despite suffering some of the deadliest disasters, disaster planning is often lacking in most low-income countries even in hospitals with some elements of a disaster plan in place, the details may not be known by key stakeholders, including the providers staffing the facility [8–11].

In Tanzania, the number of disasters has increased substantially in the past decade. These disasters have claimed the life of many citizens, leaving some with permanent disabilities, and causing disruption of infrastructure and settlement. Disaster Management activities in Tanzania are under the disaster management department in the Prime Minister’s office, and they are guided by the Disaster Relief Coordination Act, and the National Guideline and Policy for disasters [12]. The health system plays an essential role in the management of disaster. The Tanzanian public health system is a referral-based system starting at the dispensary, advancing through the health centre, the district hospital and regional hospitals, and ending at tertiary referral hospitals [13]. At the time of this study, Tanzania had 25 geo-political regions [14, 15]. The capacity and capability of the Tanzanian health care infrastructure to manage disasters is unknown. In this study, we describe the current state of disaster preparedness and response in Tanzanian regional hospitals. This will provide a baseline against which future progress regarding the impact of disaster preparedness interventions and projects can be measured and guide the development of disaster preparedness and response strategies.

## Methods

### Study design

This was a descriptive cross-sectional study of all regional hospitals in Tanzania between May 2012 and December 2012. The study was carried out as part of the Tanzania Emergency Care Capacity Site Survey project, which aimed to evaluate three main components of emergency care: disaster preparedness, equipment availability, and disease burden in all district and regional hospitals.

### Study setting and population

This study was conducted in all regional hospitals of Tanzania mainland only (excluding the islands of Zanzibar and Pemba). Tanzania is designated as a low-income country with a per capita income of around $600 US dollars, and a population of 45 million at the time of the study [16]. More than 80% of the population lives in rural areas, and a third live below the poverty line [17]. The leading causes of mortality are infectious diseases (including HIV, malaria and tuberculosis), trauma, and poorly controlled chronic medical conditions. At the time of this study, Tanzania was divided into 25 geo-political regions in the mainland, with each region having at least one referral hospital. The regional hospitals are expected to offer an expanded range of care and more specialty services than are provided at district facilities.

### Data collection and analysis

Data collection was conducted by five authors (PM, HS, JM, KM and SK) all certified medical doctors, who were each randomly assigned to assess different geographical and political areas of Tanzania, based on locations of the regional hospitals. All data collectors received training prior to starting data collection. A structured questionnaire, based on the World Health Organization (WHO) National Health Sector Emergency Preparedness and Response Tool [1], was used to interview the heads of the acute intake areas, matrons (head nurses), and medical officers in charge of each of the regional hospitals in Tanzania mainland. The questionnaire had 25 question with nine key sub-sections namely: *general information, command and control, communication, safety and security, triage, surge capacity, human resource and training, logistics, equipment and supplies, post disaster recovery*. Prior to data collection, training and testing of the questionnaire was performed. Direct observation and on-site interviews were also conducted to verify information provided during the interview.

The study data were transferred from the hand-written data forms into an Excel database (Microsoft Corporation, Redmond, WA) and analysed with SAS (version 9.3, SAS Institute Inc., Cary, NC, USA). Key outcome measures included the hospitals’ triage, communication, security, and surge capacity infrastructures. Procedure, frequency and univariate functions were performed to check for any outliers and clean the dataset. Descriptive statistics, including means, standard deviations, medians, and ranges were calculated.

## Results

### Hospital characteristics

We surveyed 25 regional hospitals (100% capture) in mainland Tanzania. There were 830 doctors affiliated with the 25 hospitals, with a median of 27 [interquartile range (IQR) 21–44) doctors per hospital. Of the 830 doctors, 352 (42.4%) were assistant medical officers (AMO), while 75 (9.0%) were specialists. There were 5390 nurses working at the 25 hospitals surveyed, with a median of 214 nurses per hospital (IQR 158–273). Majority 2061 (38.2%) of the nurses had a qualification of health attendants, while only 77 (1.4%) were nurse officers. No emergency physicians worked at any of the regional hospitals. The in-person interviews were conducted with 13-hospital doctors incharge, 9 matrons and 4 heads of casualty. Table 1.

**Table 1:** Type of personnel at Tanzanian regional hospitals

Staff type			
Doctors			
Cadre	*N* = 830	%	Median (range)
Assistant medical officers	352	42.4	12 (5–37)
Clinical officers	211	25.4	7 (1–19
General practitioners	192	23.1	4 (1–25)
Obstetrician and gynaecologists	26	3.1	1 (0–2)
Surgeons	19	2.3	0 (0–3)
Internal medicine specialist	16	1.9	0 (0–2)
Paediatricians	14	1.7	0 (0–2)
Nurses			
Cadre	*N* = 5390	%	Median (range)
Health attendants	2061	38.2	90 (18–133)
Enrolled nurses	1807	33.5	76 (9–160)
Registered nurses	1445	26.8	54 (21–112)
Nurse officer	77	1.4	2 (0–13)
Others			
Cadre	*N* = 202	%	Median (range)
Laboratory technician	114	56.4	3 (1–15)
Pharmacists	32	15.8	1 (0–4)
Pharmacy assistant	32	15.8	2 (0–6)
Laboratory technologist	24	11.9	0 (0–3)

### Disaster experience and planning in regional hospitals

In the past 5 years, 23 (92%) regional hospitals reported experiencing a disaster. As shown in Table 2, the top three causes of disasters were major road traffic crashes (MTC) 20 (87%) defined as a single event with over ten victims, floods 6 (26%), and infectious disease outbreaks 5 (22%). Three hospitals (13%) had experienced multiple casualty events resulting from bomb explosions in the past 5 years. The majority of hospitals 15 (60%) had a disaster committee, but only 5 (20%) had a disaster plan in place.

**Table 2:** Disaster experience and planning in Tanzanian regional hospitals

	*N* = 25	Percentage
Experience of disaster in past 5 years	23	92
Disaster planning		
Disaster committee	15	60
Disaster simulation	11	44
Simulation plan	5	20
Type of disaster		
MTC	20	87
Floods	6	26
Infectious disease outbreak	5	22
Plane crash	3	13
Explosions	3	13
Fire	2	9
Conflict	2	9
Landslide	1	4

### Surge capacity characteristics

Only five (20%) of the hospitals had a stockpiling area with supplies (medications and consumables onsite), though the majority (68%) had a contingency plan identifying a source for these supplies (for example a specific department or distributor designated to provide supplies during a disaster). Twenty (80%) had a contingency area for provision of care in surge situations. A temporary morgue was available in just 2 (8.3%) of the hospitals. Table 3.

**Table 3:** Surge capacity

Elements of surge capacity	*N* = 25	Percentage
Contingency treatment area	20	80.0
Contingency plan for supplies	17	68.0
Pull staff from other hospital	12	48.0
Prioritize services in disaster	8	32.0
Stockpiling area and supplies	5	20.0
Area for patient overflow	4	16.0
Temporary morgue	2	8.0

### Hospital infrastructure and equipment

All regional hospitals had electricity and a back-up generator. Intensive care was available in 11 (44%) of the hospitals. None had a computed tomography (CT) scan machine nor a decontamination area. Only 2 (8.0%) had a fire alarm system. Eighty-eight percent of hospitals were fenced, 24 (96%) hospitals had a specific entry to the hospital, and 21 (84%) of the surveyed hospitals reported controlled entry of persons into the hospital. Table 4.

**Table 4:** Infrastructure to support hospitals during disaster management

Infrastructure component	*N* = 25	Percentage
Electricity	25	100
Back-up Generator	25	100
Blood bank/refrigerator	23	92.0
Storage tanks	21	91.3
Inventory	21	91.3
Intensive care unit	11	44.0
Safety and security	N = 25	Percentage
Specific entry	24	96.0
Extinguishers	24	96.0
Fence	22	88.0
Control entry	21	84.0
Specific exit	20	80.0
Guards	20	80.0
Sand buckets	4	17.4
Fire alarm	2	8.0
Infrastructure component	Total	Median (Range)
Hospital beds	7783	350 (86–450)
Units of blood	445	20 (3–50)
Mortuary capacity	371	12 (2–50)
Wheelchairs	100	3 (2–10)
Stretchers	75	3 (1–14)
Intensive care unit beds	64	0 (0–14)
Ambulances	36	1 (0–3)
X-ray	31	1 (0–4)
Ultra sound	24	1 (0–3)
Electrocardiogram	12	0 (0–3)

### Training, triage, drills and communication

A designated triage area for everyday use was available in 10 (40%) of the hospitals. Routine sorting of patients based on the judgement of an individual provider (though without use of validated instrument) was observed in 15 (60%) hospitals. This was performed mostly by enrolled nurses (48%) or nurse attendants (44%). Only 8 (32%) regional hospitals had provided routine or surge triage training to their triage personnel.

Eleven hospitals had conducted a disaster drill in the last year, and only 5 (20%) hospitals had a plan to conduct a disaster drill in the following year. Most hospitals 24 (96%) relied on cellular phone communication during disasters. 21 (84.4%) had updated staff contacts available for use in case of need to call any available staff. The medical officer in charge acts as main contact person, linking the hospital with other stakeholders in 18 (72%) Hospitals. Only 3 (12%) hospitals had a back-up communication system. Table 5.

**Table 5:** Triage capacity and communication components available

	*N* = 25	Percentage
Triage capacity component		
Regular triage	15	60.0
Triage area	10	40.0
Triage personnel		
Triage enrolled nurse	12	48.0
Triage attendants	11	44.0
Triage Registered Nurse	5	20.0
Triage Assistant medical officer	4	16.0
Triage clinical officer	4	16.0
Triage medical doctor	1	4.0
Triage training		
Triage training	8	32.0
Triage guidelines	4	16.0
Triage forms	3	12.0
Communication component		
Mobile phone	24	96.0
Staff contacts	21	84.0
Spokesperson (Liaison)	18	72.0
Command centre	10	40.0
Landline phone	9	36.0
Conference area	7	28.0
Siren	4	16.0
Back-up communication	3	12.0

## Discussion

This study represents one of the most comprehensive surveys of regional hospitals in sub-Saharan Africa (SSA), a region with one of the highest rates of conflicts, natural emergencies and disruption of services [18]. Our results show that nearly all-regional hospitals experienced a disaster in the past 5 years, further demonstrating the importance of preparedness to ensure resilience to emergencies and disasters. Disasters reported were most often caused by large MTCs (87%). This finding is consistent with prior studies, which have shown an increase in MTCs in Tanzania due to rapid urbanization, deficient road conditions and poor adherence to general road safety [19–21].

We have noted several gaps in disaster preparedness in Tanzanian regional hospitals. Human resources available for health care delivery at each regional hospital are below the recommended ratio for all the cadres [22]. Similar to prior studies done in SSA [23], we found the few highly skilled workers tended to be in administrative positions at the hospital, which limited their clinical roles. Thus, when disasters occur, responding personnel might be junior clinical or nursing staff. In our study, the Assistant Medical Officers and Clinical Officers formed the largest group of clinicians in regions that were remotely located and under-resourced; whereas specialists and medical officers were more prevalent in big cities. This uneven distribution suggests the need to re-distribute the workforce as the numbers of medical officers and specialists increase, so as to improve the capacity of regional hospitals to respond to disasters.

Another gap identified was the lack of disaster planning in more than half of the regional hospitals. Forty percent of the hospitals had no disaster committee at all. Disaster plans and a disaster committee are paramount to effective management of any disaster [23, 24] as they lay out a clear plan for how to effectively address disaster-related challenges and delineate the roles and required resource allocation during a disaster.

The review of elements to support catastrophic surge revealed that no hospital had all components of surge capacity. Further analysis showed that 84% of hospitals had fewer than 50% of the surge capacity components. Furthermore, close to one-half of the hospitals reported the ability to pull in staff from other facilities in a disaster. We believe this is a result of similar phenomenon observed in previous studies in Tanzania [24, 25], which noted the over-saturation of hospital beds with very sick patients, a situation which significantly stretches providers capacity at baseline, resulting in lack of additional staff to mobilize during a disaster. Prior studies recommended that for a hospital to be capable of taking care of patients in disasters, it should be able to expand its operations for both paediatrics and adults to about 500 patients per million population [26, 27]. In Tanzania, this would require increasing capacity to treat approximately an additional 22,000 patients. To address a catastrophic surge with limited staff and resources, a number of actions have been proposed as being effective in supporting the disaster response and mitigating morbidity and mortality [28, 29]. Such actions include discharging stable patients from emergency departments and hospitals, cancelling elective surgeries, opening alternate care areas, and calling in stand-by or off-duty staff. However, all these approaches require careful pre-event planning.

Regional hospitals in Tanzania have one x-ray machine on average, and therefore their capacity to handle casualties requiring diagnostic radiography is limited to about six patients an hour [27]. This can cause a large delay or inadequate care of patients in event of a mass casualty incident. ICU beds are available in less than half of the hospitals, and while our study was not designed to assess ICU capacity, previous studies from similar settings have shown variable and poor levels of resources available in most Tanzanian ICUs, limiting the capacity to care for critically ill patients [30]. All regional hospitals have electricity, back-up generators and wheel chairs; however, none had CT scan machines, reflecting high variability in elements available to support hospitals during disasters.

Triage is a crucial component of routine emergency care and of disaster management [31]. In our survey, more than half of the hospitals reported having a “triage system” in place, though most of these referred to having a clinical provider sort patients based on individual judgement not to use of a validated instrument or systematic protocol. Further more, less than one third of providers involved in triage had received training. During a disaster event, the mass influx of people in a hospital is likely to add stress to an already overextended hospital staff. Providers may be pulled from clinical care to attend to their own family members, political leaders, media personnel, and the non-critical patients [32]. validated triage protocols and training are necessary to ensure effective care and appropriate resource utilization. While we did not directly assess the knowledge and practices of hospital staff our findings suggests a potential gap in emergency preparedness and response capability of hospital staff and future studies should focus on studying and addressing this gap. Safety and security for staff are also necessary to enable care for patients. Most regional hospitals are fenced with a designated entry, which makes it possible to control entry into the hospital compounds. However, the majority of hospitals did not have a fire alarm system and none has a decontamination area. Communication was found to rely mainly on cellular network phones and landline telephones which have been shown to fail due to overwhelming volume in disasters [33, 34]. Disasters are likely to overwhelm communication networks within the facility and outside. It is therefore important to have a back-up communication system or facility-specific plan such as radios and runners.

### Limitations

Some of our results are based on reported rather than observed data and this may limit accuracy; however we believe that this has limited impact as all interview subjects were lead administrators. We did not measure the vulnerability of the hospitals and early warning systems for disaster in each region, but our results provide a baseline against which future studies can build on. Data were also collected during a brief visit and may not reflect conditions year round though this effect is likely to be limited as we report on facility characteristics without high seasonal variation.

## Conclusion

This nationwide survey found that hospital disaster preparedness is at an early stage of development in Tanzania, and important opportunities exist to better prepare regional hospitals to respond to disasters. We have identified specific areas for potential action based on our findings. We hope that our findings and discussion will support coordinated planning at the regional and national level in Tanzania.

## Abbreviations

AMO: Assistant Medical Officer
CDC: Centre for Disease Control
CO: Clinical Officer
GP: General Practitioner
ICU: Intensive care Unit
MSD: Medical stores department
MUHAS: Muhimbili University of Health and Allied Sciences
NGO: Non Governmental Organization
RBG: Random Blood Glucose
TANESCO: Tanzania Electricity Supply Company
